# *Southern African Journal of HIV Medicine* August 2021

**DOI:** 10.4102/sajhivmed.v22i1.1309

**Published:** 2021-10-15

**Authors:** David C. Spencer

**Affiliations:** 1 Division of Infectious Diseases, Faculty of Medicine, University of the Witwatersrand, Johannesburg, South Africa

## Special collection: UNAIDS targets for 2030

HIV/AIDS in Southern Africa. An end to new HIV infections by 2030? The views of our authors. Summaries of recent articles in the *Southern African Journal of HIV Medicine* (SAJHIVMED).

Despite the passage of three decades, people living with HIV in South Africa are still at risk of serious morbidity and inappropriate mortality from HIV. In order to achieve the target of ending HIV by 2030, a more urgent public health response is required. This must include more innovative strategies to improve HIV awareness, new thought with regard to prevention, upgrading of ART services and a renewed dedication to the retention in care of all people living with HIV.^1^

If significant improvements in differentiated service delivery, increases in human resources and HIV prevention can be realized, Botswana could become one of the first countries with a previously high-burdened generalized HIV epidemic to gain epidemic control, despite the demands of the COVID-19 [*coronavirus disease 2019*] pandemic.^2^

This year marks the 21st anniversary of the existence of the SAJHIVMED. The journal was first published in 2000. At the time, HIV denialism was rampant, many were confused and large numbers died unable or unwilling to accept or access antiretroviral treatment (ART). At the time, the Southern African HIV Clinicians’ Society believed that a journal of HIV medicine was necessary to showcase the work of local researchers and to underpin the southern African HIV epidemic with credible science. Though times have changed, the mandate has not. There is much talk of an end to new HIV infections by 2030. My thanks to the guest authors who have submitted articles to the journal addressing this topic. Like Dorothy,^3^ ‘we’re not in Kansas anymore!’ We are not where we were. Antiretrovirals have changed the epidemic. Still no protective vaccine exists. And no cure has been found. If there is a rainbow at the end of our story, it is not yet in sight. We are in Africa. And the task ahead – an end to new HIV infections by 2030 – is still far off. In the following pages, I summarise several SAJHIVMED articles that address this story. If you have the opportunity, please read the parent articles. These can be accessed through the links provided at the end of each citation. COVID-19 has demonstrated the vulnerability of our world – particularly the low- and middle-income countries. Climate change is here. We have an uncertain future on this planet. Substantial fault lines have emerged in South African society in recent days. Here, too, the winds of change are blowing. As healthcare workers, it is time to do what we can to heal our world and the angry communities around us. We are the so-called experts. All of us seek an end to this HIV epidemic.


**David C. Spencer**
Editor-in-Chief
SAJHIVMED


## Summaries of scientific articles published in the 2021 edition of the SAJHIVMED

1 Parker E, Judge MA, Macete E, et al. HIV infection in eastern and southern Africa: Highest burden, largest challenge, greatest potential. S Afr J HIV Med. 2021;22(1):a1237. https://doi.org/10.4102/sajhivmed.v22i1.1237

**Summary:** The authors review the HIV epidemic in eastern and southern Africa (ESA) in the light of the failure of most countries in the region to meet the 2020 UNAIDS 90-90-90 targets and the call to meet the new UNAIDS targets by 2030 (See the full article for the UNAIDS 2020, 90-90-90 targets).

The UNAIDS 2025–2030 targets include the following: ≥ 95% of those living with HIV to know their HIV status, ≥ 95% of this group to have started and remain on antiretroviral therapy (ART) and ≥ 95% of those on treatment to have persistently undetectable viral loads by 2025–2030.

‘Know your epidemic, know your response’ (Wilson et al. 2008, Further reading). Our authors argue that a new commitment and new targets are needed for the current decade. A total of 20.7 million, or 54%, of the globe’s 38 million people living with HIV live in ESA. Most (87%; range: 15% – 98%) are aware of their diagnosis, 83% (37% – 98%) are on ART, and 90% (68% – 97%) of these have suppressed viral loads. Interpret the numbers with caution: ranges and confidence intervals are wide, the background data is incomplete, and many clinics and individuals are likely to have been missed. The problems include the following:

- *Retention in care*. Africa’s men are still too easily lost from care.
- *Infrastructure*. There are too few high-throughput labs and insufficient point-of-care tests and testing.
- *The neglected*. How well represented are our key populations and are these being adequately reached? Our men who have sex with men, those who inject drugs, female sex workers, the truck drivers, the migrants, mobile miners, the serodiscordant couples, pregnant women and their infants? Why are these so often missed?

The authors question whether these key groups are on the 2030 roadmap. To this, they add the ‘acutely infected’ – a high-risk transmission group. How well are these groups being recognised and targeted in ESA? The authors draw attention to multiple gaps – multiple opportunities – throughout ESA. This is an important article. It asks pertinent questions and gives some answers:

- Promote and expand local *prevention* research (and implementation).
- Improve *accessibility* to HIV education and testing.
- Ramp up in-country *point-of-care* clinical trials of affordable and available viral load and CD4 testing diagnostics.

Read the article. In particular, look at the figures, especially Figure 1b (See link: https://doi.org/10.4102/sajhivmed.v22i1.1237) South Africa’s HIV numbers dwarf its regional neighbours’. ‘Highest burden, largest challenge, greatest potential’. Do we want a world without new infections by 2030? Yes. Yes. Yes. However, as a region, we are languishing far behind target.

### Further reading

- Wilson D, Halperin DT. ‘Know your epidemic, know your response’: A useful approach if we get it right. Lancet 2008;372(9637):423–426. https://doi.org/10.1016/S0140-6736(os)60883-1

2 Pillay Y, Johnson L. World AIDS Day 2020: Reflections on global and South African progress and continuing challenges. S Afr J HIV Med. 2021;22(1):a1205. https://doi.org/10.4102/sajhivmed.v22i1.1205

**Summary:** With regard to eliminating new HIV infections by 2030, where are we? Three 2020 reports are reviewed: UNAIDS (global), the HIV Policy Lab (global) and Thembisa version 4.3 (local, South African) (Further reading: UNAIDS 2020; HIV Policy Lab 2020; Johnson 2020). Table 1 in this article outlines South Africa’s 2020 90-90-90 goal attainment by province, gender and age (follow the link above to see the article and the table in the SAJHIVMED). *Adult females do best* (94-74-92). This is followed by adult males (91-67-92) and lastly children (79-70-72). An important detail is recorded in the table’s columns: only 54% of adult males living with HIV in the North West province have been diagnosed. Only 58% of children living with HIV in the province of Limpopo have been diagnosed and are on ART! In Gauteng, the economic hub of South Africa, only 66% of children living with HIV are on ART – and only 63% are virologically suppressed!:

Five years into the implementation of [*South Africa*] Sustainable Development Goals SDG [*Sustainable Development Goals*], our children lag behind their adult counterparts. Urgent action is called for. (Nyasulu et al. 2021, see Further reading)

The article provides the reader with numbers. Between 2010 and 2019, South Africa’s total HIV-infected population increased from 5.9 to 7.64 million. In the same period, the overall HIV incidence rate fell by 55%. New infections still occur: 201 000 in 2018/2019. However, the percentage of ‘ever-tested’ South Africans has risen, from 47.3% in 2010 to 76.3% currently. Has there been improvement? Yes. However, we need more … please.

**Prevention?** Politicians, health administrators and regulatory bodies, industry and healthcare workers *must* make it easier for *all* South Africans to access antiretrovirals and care. ‘U=U: an undetectable viral load on ART = an untransmissible virus’ (Further reading: Thigpen et al. 2012). In modern practice, ART is both treatment and prevention. However, condom use at ‘last sex’ among 15–24-year-old South Africans was 23% in 2012 and was still only 29% in 2019. Among pregnant Sowetan women, HIV prevalence rates have not changed in two decades: 28.9% in 2002, 33.1% in 2009 and 27.4% in 2015 (Further reading: Mnyani 2020). And in 2019, the national South African antenatal HIV prevalence rate was 30.7% (confidence interval [CI], 30.4% – 31.3%) (Further reading: Woldesenbet et al. 2019).

Can the prevention gap be closed? Pre-exposure prophylaxis (PrEP) represents an underutilised opportunity. Only 3% of South African female sex workers and 1% of men who have sex with men use PrEP regularly. Rates in the general population are similarly low; PrEP reliably protects the uninfected. The SAJHIVMED’s 2020 PrEP guidelines speak to its efficacy, safety and affordability (Further reading: Bekker et al. 2020). Every clinician in Africa must become familiar with these guidelines and ensure that all are protected. The at-risk groups are known, but with a countrywide HIV prevalence of 19.7% among 15–49-year-old South Africans, virtually all are at risk. Long-acting antiretroviral (ARV) injectables represent a new day in HIV prevention. Data from the HPTN 083 and HPTN 084 cabotegravir studies confirm PrEP’s utility (efficacy) in Africa and its value in the poorly adherent (Further reading: Clement et al. 2020; Landovitz et al. 2020; Delany-Moretlwe et al. 2021). Looking to 2030, long-acting PrEP must be a front-runner.

Has the COVID-19 pandemic taught us lessons that may help us achieve the UNAIDS 2030 goals?:

- *Leadership*. Competent, respected, visible and committed to an HIV-free society.
- *A defined strategy and a defined target population*. Be strategic and inclusive. Acknowledge that the individual is important. Provide differentiated care. Address stigma. Make HIV clinics accessible and welcoming. We need new thoughts on adherence: where should we be placing the long-acting ARVs?
- *Aim to reduce harm*. Antiretrovirals reduce harm; we need to involve key groups, providing ART for the infected and PrEP for the uninfected: *ART is for the entire community*. Retention in care for the infected. Push the 95-95-95 agenda.
- *In-your-face and regularly updated science-based HIV education.* Promote the 2030 targets. Keep all informed, with a regular slot on every television and radio channel. Keep the foot on the accelerator.
- *Involve the community*. We are all in this together. Close the gaps.

### Further reading

- UNAIDS. Prevailing against pandemics by putting people at the centre [homepage on the Internet]. Geneva; 2020 [cited n.d.]. Available from: https://www.unaids.org/sites/default/files/media_asset/prevailing-againstpandemics_en.pdf
- HIV Policy Lab. 2020 global HIV policy report: Policy barriers to HIV progress [homepage on the Internet]. 2020 [cited n.d.]. Available from: https://www.hivpolicylab.org/documents/reports/2020GlobalReport/2020%20HIV%20Policy%20Lab%20Executive%20Summary%20
- Johnson LF, Dorrington RE. Thembisa version 4.3: A model for evaluating the impact of HIV/AIDS in South Africa [homepage on the Internet]. 2020 [cited n.d.]. Available from: https://www.thembisa.org/
- Nyasulu JCY, Maposa I, Sikakhane BP, Pandya H. Access to HIV services and viral load suppression among children during the 90-90-90 strategy implementation in South Africa: A time-series analysis. S Afr J HIV Med. 2021;22(1):a1187. https://doi.org/10.4102/sajhivmedv22i1.1187
- Thigpen MC, Kebaabetswe PM, Paxton LA, et al. Antiretroviral preexposure prophylaxis for heterosexual HIV transmission in Botswana. N Engl J Med. 2012;367(5):423–434. https://doi.org/10.1056/NEJMoa1110711
- Mnyani C, Tait CL, Peters RPH, et al. Implementation of a PMTCT programme in a high HIV prevalence setting in Johannesburg, South Africa: 2002–2015. S Afr J HIV Med. 2020;21(1):a1024. https://doi.org/10.4102/sajhivmed.2020.v21i1.1024
- Woldesenbet SA, Kufa T, Lombard C, et al. The 2017 national antenatal sentinel HIV survey key findings, South Africa [homepage on the Internet]. [cited 2021 Sept 22] Available from: nicd.ac.za/wp-content/uploads/2019/07/Antenatalsurvey-report-24July19.pdf
- Bekker LG, Brown B, Joseph-Davey D, et al. Southern African guidelines on the safe, easy and effective use of pre-exposure prophylaxis: 2020. S Afr J HIV Med. 2020;21(1):a1152. https://doi.org/10.4102/sajhivmed.v21i1.1152
- Clement ME, Kofron R, Landovitz RJ. Long-acting injectable cabotegravir for the prevention of HIV infection. Curr Opin HIV AIDS. 2020;15(1):19–26. https://doi.org/10.1097/COH.0000000000000597
- Landovitz R, et al. HPTN 083 final results: pre-exposure prophylaxis containing long acting cabotegravir is safe and highly effective for cis-gender man and trans-gender women who have sex with men. International AIDS Conference (IAC); July 6–10, 2020 [cited 2021 Jan 29]. Available from: https://llwww.natap.org/2020/IAC/IAC_28.htm
- Delany-Moretlwe S, Hughes JP, et al. Long acting cabotegravir is safe and effective in preventing HIV infection in cisgender women: Interim results from HPTN 084, HIV R4P. HIV Research for Prevention conference - Virtual, January 27-28 and February 3-4, 2021. HY01.02.

3 Meya DB, Tugume L, Nabitaka V, et al. Establishing targets for advanced HIV disease – A call to action. S Afr J HIV Med. 2021;22(1):a1266. https://doi.org/10.4102/sajhivmed.v22i1.1266

**Summary:** The World Health Organization (WHO) defines advanced HIV disease (AHD) as HIV-associated disease in persons living with HIV and presenting to care with a CD4 cell count of < 200 cells/µL or with a WHO Stage 3 or 4 infection. The definition includes *all* children under the age of 5 years irrespective of their CD4 count (Further reading: WHO 2017). Advanced HIV disease carries a high mortality and is more frequent in the hospitalised (Further reading: Carmona et al. 2018; Laher et al. 2021).A third of those who enter or who cycle in and out of HIV care have AHD. Many are ART experienced. *Can AHD be harnessed by 2030?* The 95% UNAIDS-2030 targets are a strategic opportunity (Meya et al. 2021 above):

[*W*]e suggest that these specific targets … CD4 testing, CrAg [*cryptococcal antigen*] and *TB* testing, and treatment … aligned to the WHO AHD package of care would be a step in the right direction.

The authors suggest a focus on:

- the newly HIV diagnosed
- those previously in care and (now) returning to care
- those failing antiretroviral treatment.

Identify persons with CD4+ counts of < 200 cells/µL, and prioritise checking the serum CrAG lateral flow assay and urine Xpert/tuberculosis (TB) lipoarabinomannan (LAM). Institute rapid ART initiation if patients are ART naïve or returning to or failing care. Always anticipate TB and TB-associated immune reconstitution – *never let it surprise you*. The writers urge the strengthening of measures to track and retain patients in care throughout sub-Saharan Africa.

Much in this article has been said before. However, this is a timely call to Africa’s healthcare community to do something memorable: close the door on HIV by 2030. Is it doable? Read the article.

### Further reading

- WHO. Guidelines for managing advanced HIV disease and rapid initiation of antiretroviral therapy. Geneva: World Health Organization; 2017.
- Carmona S, Bor J, Nattey C, et al. Persistnet high-burden of advanced HIV disease among patients seeking care in South Africa’s National HIV Programme: Data from a nationwide laboratory cohort. Clin Infect Dis. 2018;66(Suppl_2):S111–S117. htpps://doi.org/10.1193/cid/ciy045
- Laher AE, Venter WDF, Richards GA, Paruk F. Profile of presentation of HIV-positive in-patients to an emergency department in Johannesburg, South Africa. S Afr J HIV Med. 2021;22(1):a1177. https://doi.org/10.4102/sajhivmed.v22i1.1177

4 Laher AE, Venter WDF, Richards GA, Paruk F. Profile of presentation of HIV-positive patients to an emergency department in Johannesburg, South Africa. S Afr J HIV Med. 2021;22(1):a1177. https://doi.org/10.4102/sajhivmed.v22i1.1177

**Summary:** This observational study examines the demographics, clinical presentation, diagnosis and outcome of 1224 persons living with HIV (PLWH) consecutively admitted to the emergency department of a large public hospital in Johannesburg between July 2017 and October 2018. Of these, 17.3% were ART naïve. Of the 75.2% who had started ART prior to this admission, 32.2% reported non-adherence.

This is a description of South Africans who sought medical help when sick – for some, too late. Although the group is young (median age, 36 years; interquartile ratio [IQR], 31–44 years), in-hospital mortality was high (*n* = 166; 13.6%). Immunity often severly weakened: the median CD4 count was 112 (IQR, 34–295) cells/µL, and almost half, *n* = 527 (47.5%), had a baseline CD4 of < 100 cells/µL. Active TB was diagnosed in 244 (20%), and 213 (86.3%) had extrapulmonary or disseminated TB infection.

The study has limitations: it is observational and its generalisability is limited. A priori, it has an emergency room bias that leans towards severity and poorer outcomes. Nevertheless, the authors draw a credible picture of the practice of everyday medicine in South Africa’s public sector. Their article asks, ‘[*w*]hy the late presentation? Why the break with prior antiretroviral care?’

It is a timely story with which to address the call for an end to new HIV infections by 2030. The authors remind the reader that (Laher et al. 2021 above):

[*T*]wo-thirds of the global population of people living with HIV (PLWH) are in sub-Saharan Africa (SSA). South Africa (SA) contributes approximately 7.5 million to the global number, more than twice that of any other country worldwide … the burden of HIV-related illness is still substantial, especially among the newly diagnosed, ART-naïve and those recently initiated onto treatment.

This article speaks to the practice of medicine in South Africa. It deserves to be read widely, as a reminder that HIV medicine is also about caring for the sick. I ask myself, what went wrong? Why are PLWH still dying in emergency room wards?

5 Archary M, Van Zyl R, Sipambo N, Sorour G. Optimised pediatric antiretroviral therapy to achieve the 95-95-95% goals. S Afr J HIV Med. 2021;22(1):a1278. https://doi.org/10.4102/sajhivmed.v22i1.1278

**Summary:** Eighty-four percent of new global HIV infections in children in 2019 were in Africa. The first half of this opinion piece reviews South Africa’s experience in meeting the 2020 UNAIDS 90-90-90 targets in children.

South Africa’s children are falling behind. The achieved overall targets in children were 79-47-34! While peripartum and pregnancy-related mother-to-child transmission in South Africa is now uncommon, breastfeeding-related transmission (4.3%) continues. And children infected in infancy still slip through the net to reappear, treatment naïve, in late childhood or adolescence. The second half of the article discusses advances in the management of children living with HIV in South Africa. Medication, adherence and ‘responsible care’ are nonetheless persistent points of worry, as are health systems. The authors cite the 7-year delay between registration of an essential fixed-dose paediatric generic-combination antiretroviral by the Food and Drug Administration in the United States and registration in South Africa.

The good news in this report is in the new drug formulations for the very young: a dispersible, scored combination of abacavir/lamivudine (120/60 mg) for children weighing from 3 kg to 25 kg and a dispersible, scored 10 mg dolutegravir tablet to treat children from 4 weeks of age or weighing > 3 kg. These await approval by the South African Health Products Agency (SAHPRA). Future innovations? The long-acting injectables (8-weekly cabotegravir/rilpivirine) for children and adolescents aged 12–18 years. Will these be the answer to the current unimpressive viral suppression rates?

What about the larger context of children’s health on our subcontinent? Consider that in the first year of the COVID-19 epidemic, 23 000 teenage (10–19 years) pregnancies occurred in South Africa’s Gauteng province, of which 934 were in girls aged 10–14 years (Further reading: Bengu 2021). Gender-based violence takes many forms. This is one. The subtext of this article is exactly that society’s children, particularly those living with HIV, deserve better. Children lag behind. The childhood gap in the diagnosis of HIV, treatment and viral suppression must be closed before 2030.

### Further reading

- Bhengu L. Gauteng records more than 23 000 teen pregnancies in one year, some moms as young as 10. News24 , 2021 August 17.

6 Jefferis K, Avalos A, Phillips H, et al. Five years after treat-all implementation: Botswana’s HIV response and future directions in the era of COVID-19. S Afr J HIV Med. 2021;22(1):a1275. https://doi.org/10.4102/sajhivmed.v22i1.1275

**Summary:** This review of Botswana’s current and projected response to the HIV epidemic is clear-headed, thoughtful, data based and, in the main, optimistic. It details the country’s roadmap from 2016 to the anticipated goal of no new or incident HIV infections by 2030. Unlike most of Africa, Botswana achieved the 90-90-90 UNAIDS targets before 2020! The country’s population (2.4 million) is small and mostly urbanised: 375 900 (IQR, 353 500–400 150), or 15.7%, live with HIV.

Results from the current Botswana AIDS Incidence Survey are due in 2022. The expectation is that these will validate the country’s positive trajectory towards the 2030 UNAIDS goals and perhaps achieve these before that date! Some issues remain. Women aged 15–24 years are at great risk of infection. Innovative, targeted ideas around prevention are needed. Many ‘first-time’ ART initiates (25%) do so with a CD4 count of < 200 cells/µL, that is, with advanced immune suppression. And many (76%) with CD4 counts of < 200 cells/µL are actually ‘ART experienced’, some poorly adherent and failing treatment and others with a dysfunctional immune response and a suppressed viral load. This represents a group needing more attention.

In their economic assessment, the authors anticipate a contraction of gross domestic product as a result of COVID-19. Botswana’s government has had to increase spending. Budget deficits have been unavoidable. However, public spending is still expected to fill the gaps.

Looking to 2030, the authors define what is still needed:

- greater investment in sexual reproductive health interventions such as PrEP, particularly for young women
- national and international advocacy to reduce the exorbitant costs to Africa of laboratory reagents, commodities and supplies
- strengthening of differentiated care to those living with HIV and the streamlining of the care of those stable on minimal intervention (e.g. fewer laboratory tests)
- the upskilling (capacitation) of healthcare workers to identify and manage patients with AHD.

This article is a hopeful look at Botswana’s future. It is recommended reading.

7 Lilian RR, Davies N, Gilbert L, et al. CD4 testing after initiation of antiretroviral therapy: Analysis of routine data from the South African HIV programme. S Afr J HIV Med. 2020;21(1):a1165. https://doi.org/10.4102/sajhivmed.v21i1.1165

**Comment**: I have included this article with our 2021 articles looking at achieving the UNAIDS 2030 guidelines because it speaks to the role of the CD4 cell count in long-term HIV care. This, too, has bearing on achieving the 2030 goals.

The authors follow the trajectory of the CD4+ cell count post-ART initiation in a large anonymised electronic South African patient database, the Three Interlinked Electronic Registers project, or TIER.Net. Records are from 2004–2021 and describe 1 178 190 persons in the South African public sector on ART in two urban (Gauteng) and two rural (Limpopo) settings. Overall, baseline CD4+ cell counts were low: 50% had CD4+ counts of < 200 cells/µL. By 2017, this percentage had improved to 37.2%. However, only 46.5% of CD4 counts captured on TIER.Net were repeated. Of these, 14.3% PLWH (*n* = 78 494) remained with CD4 counts of < 200 cells/µL. Indeed, 20% (*n* = 18 566) of those on ≥ 4 years of ART *and* with viral suppression, viz. a viral load (VL) of < 1000 copies/mL, were immune-non-responders or immune-discordant responders (Further reading: Yang et al. 2020; Laprise et al. 2013). The latter were likely to be on second-line ART (adjusted odds ratio [aOR], 1.79), older, viz. 35–45 years and particularly, > 45 years (aOR, 1.15 and 1.50, respectively), male (aOR, 2.28) and to have confirmed tuberculosis (aOR, 2.49). *Baseline* CD4 cell counts of > 350 cells/µL were protective of long-term immune deficiency (aOR: 0.35)!

So, when should CD4 tests be repeated in those on ART? ‘Every 6 months post-baseline if the preceding CD4+ count was < 200 cells/µL’. However (Further reading: Nel et al. 2020):

[*I*]f the CD4 count is > 200 cells/µL at baseline or it increases above this threshold on ART, then CD4 testing can be stopped, as therapeutic monitoring of ART is best accomplished with VL, not with CD4 count or clinical criteria. The efficacy of ART is measured not with repeated CD4 cell counts, but with regular VL monitoring.

Beyond the mechanics of treating those with a persistently low CD4+ count, Lilian et al.’s article is asking a bigger question: Does the immune system *ever* fully recover from HIV? What does this mean for the future? We are not at the end of the chapter on HIV. Nor do we fully understand what lies ahead.

This is an article that should be read and its message ruminated on.

### Further reading

- Yang X, Su B, Zhang X, et al. Incomplete immune reconstitution in HIV/AIDS patients on antiretroviral therapy: Challenges of immune non-responders. J Leuko Biol. 2020;107(4):597–612. https://doi.org/10.1002/JLB.4MR1019-189R
- Laprise C, De Pokomandy A, Baril J-G, et al. Virologic failure following persistent low-level viremia in a cohort of HIV-positive patients: Results from 12 years of observation. Clin Infect Dis. 2013;57(10):1489–1496.
- Nel J, Dlamini S, Meintjes G, et al. South African HIV clinicians’ society guidelines for antiretroviral therapy in adults: 2020 update. S Afr J HIV Med. 2020;21(1):a1115. https://doi.org/10.4102/sajhivmed.v21i1.1115
